# Targeting TR4 nuclear receptor suppresses prostate cancer invasion *via* reduction of infiltrating macrophages with alteration of the TIMP-1/MMP2/MMP9 signals

**DOI:** 10.1186/s12943-014-0281-1

**Published:** 2015-01-27

**Authors:** Xianfan Ding, Dong-Rong Yang, Liqun Xia, Bide Chen, Shicheng Yu, Yuanjie Niu, Mingchao Wang, Gonghui Li, Chawnshang Chang

**Affiliations:** Department of Urology and Chawnshang Chang Liver Cancer Center, Sir Run Run Shaw Hospital, School of Medicine, Zhejiang University, Hangzhou, 310016 China; George Whipple Lab for Cancer Research, Departments of Pathology, Urology and Radiation Oncology, and The Wilmot Cancer Center, University of Rochester Medical Center, Rochester, NY 14646 USA; Department of Urology, the 2nd Affiliated Hospital of Soochow University, Suzhou, 215004 China; Chawnshang Chang Sex Hormone Research Center, Tianjin Institute of Urology, Tianjin Medical University, Tianjin, 300211 China; Sex Hormone Research Center, China Medical University/Hospital, Taichung, 404 Taiwan

**Keywords:** TR4, Macrophage, Prostate cancer, Invasion, TIMP-1

## Abstract

**Background:**

TR4 nuclear receptor 4 (TR4) plays an important role in macrophages-associated foam cell formation of cardiovascular diseases and infiltrating macrophages are critical for prostate cancer (PCa) progression. However, the linkage of macrophages and TR4 and their impacts on PCa metastasis remains unclear.

**Results:**

Knocking-down TR4 in human PCa cells (C4-2, CWR22Rv1), but not in human macrophages cells (THP-1), led to suppress the macrophages infiltration to PCa cells. The consequences of such suppression of the recruitment of macrophages toward PCa then resulted in suppressing the PCa cell invasion. Mechanism dissection found that knocking-down TR4 in PCa cells suppressed metastasis-related genes including MMP2, with induction of TIMP-1. Interruption assays using TIMP-1 neutralizing antibody could then reverse TR4-macrophage-mediated PCa invasion. IHC staining showed higher TR4 level, more macrophage infiltration, lower TIMP-1 and stronger MMP2/MMP9 in tumor tissues of the Gleason score 5 + 4 patients compared with the Gleason score 3 + 3 patients.

**Conclusion:**

Targeting TR4 in prostate tumor microenvironment might represent a potential new therapeutic approach to better battle PCa metastasis.

**Electronic supplementary material:**

The online version of this article (doi:10.1186/s12943-014-0281-1) contains supplementary material, which is available to authorized users.

## Background

Prostate cancer (PCa) progression may be influenced by many cells co-existing in the tumor microenvironment that includes several types of immune cells [1-4]. These infiltrating immune cells may secrete various cytokines or chemokines to affect the PCa progression. In return, PCa cells may also be able to secrete different cytokines or chemokines to influence the migration of these infiltrating immune cells toward PCa. The consequences of these mutual interactions between PCa cells and infiltrating immune cells may then alter the PCa cell proliferation, invasion and survival [5-8]. Among many infiltrating immune cells, macrophages have recently been demonstrated to play important roles to promote PCa initiation [1] and invasion [2,3].

Testicular nuclear receptor 4 (TR4) is a member of the nuclear receptor superfamily that functions as a transcriptional regulator to modulate its downstream target genes for its influence on selective diseases [9-13] including PCa [14], metabolic syndrome and cardiovascular diseases [15-18], as well as aging [19], cerebellar development [9], and fertility [20,21]. Importantly, TR4 could also play a critical role in macrophages-associated foam cell formation of cardiovascular diseases [22] and mycobacterium tuberculosis disease [23]. However, the linkage of macrophages and TR4 and their influence on PCa metastasis remains unclear.

Here we applied the co-culture invasion system with human macrophage THP-1 cells and human castration resistant prostate cancer (CRPC) C4-2 and CWR22Rv-1 cells to demonstrate that targeting TR4 *via* TR4-siRNA suppresses the recruitment of infiltrating macrophages and the consequences of such suppression may then lead to inhibit the Pca invasion.

## Results

### Knocking-down TR4 alters the macrophage migration in PCa-macrophages co-culture system

We first applied the *in vitro* macrophage recruitment assays with co-culture of human PCa C4-2 cells and human THP-1 monocytes/macrophages to study the effect on macrophage recruitment to PCa cells. We collected three different conditioned media(CM) from C4-2 cells cultured alone, THP-1 cells cultured alone and C4-2 and THP-1 co-cultured, and results showed the CM from co-cultured C4-2 and THP-1 cells had stronger influence on recruiting THP-1 cells (Additional file 1: Figure S1).

**Figure 1:**
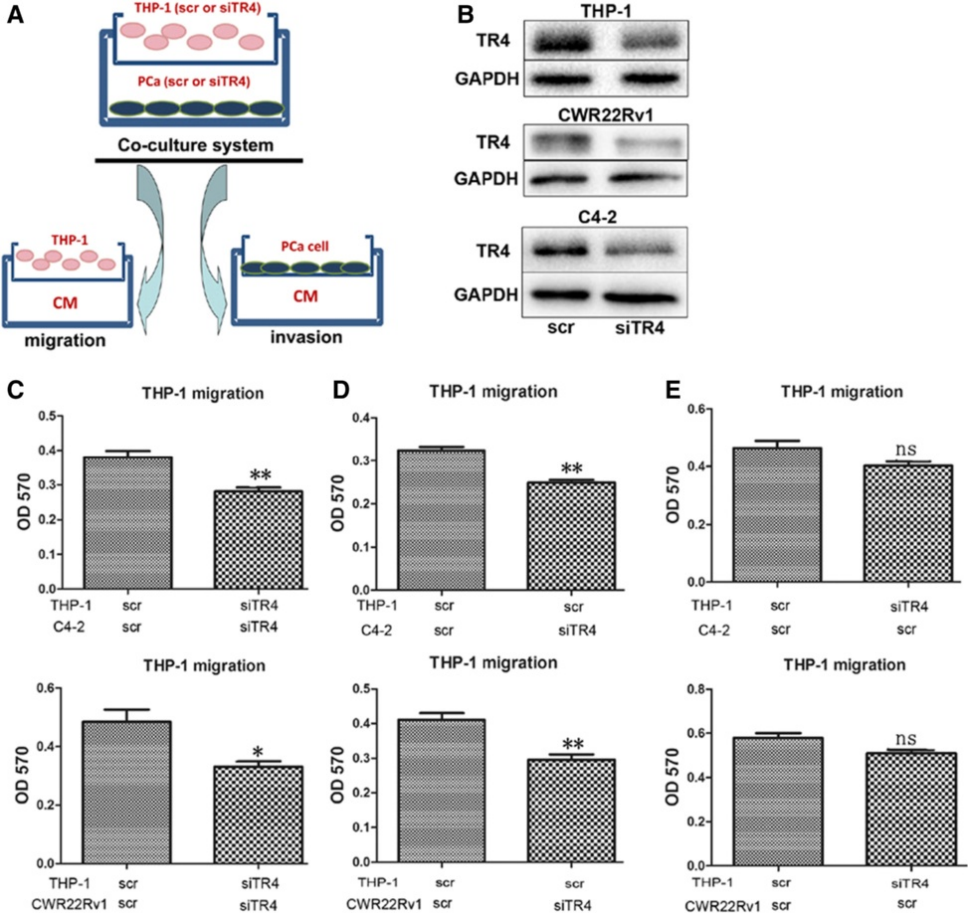
**Knocking down TR4 reduces macrophage recruitment to PCa cells in co-culture system using Transwell assays. A**. The illustration of macrophage recruitment (migration) and PCa invasion (invasion) *in vitro* model. The PCa cells were placed on the upper chamber and THP-1 cells in the lower chamber, co-cultured for 24 hours and then four kinds of conditioned media (CM) were collected: PCa scr/THP-1 scr, PCa siTR4/THP-1 scr, PCa scr/THP-1 siTR4, PCa siTR4/THP-1 siTR4. The CMs were diluted with 10% FBS RPMI media at 1:1 ratio, then put into the lower chamber of other transwell plates for macrophage migration assays and PCa cells invasion assays. **B**. The manipulation of TR4 in PCa cells and THP-1 cells. Western blotting to show the knock-down efficiency of TR4 in C4-2 and CWR22Rv-1 PCa cell lines and THP-1 macrophage cell line. **C-E**. Macrophage recruitment under different CMs. Upper are CM from C4-2/THP-1 cells and lower are from CWR22Rv1/THP-1 cells. Knock-down of TR4 in both PCa and THP-1 cell lines is shown in the left panels, knock-down of TR4 in PCa cells alone shown as **D** and knock-down of TR4 in THP-1 cells alone shown in as **E**. Note: * P < 0.05; ** P < 0.01; ns P > 0.05.

The PCa scr (scramble)/siTR4 cells were co-cultured with THP-1 scr/siTR4 cells (Figure 1A, upper) respectively for 24 hours in the 0.4 μM transwell plates, and CMs were collected and diluted with regular media before applying in the human THP-1 macrophage recruitment assay in the 5 μM transwell plate system (Figure 1A, lower and left). The manipulation of TR4 protein levels were shown in Figure 1B.

The results revealed that the CM from the PCa siTR4/THP-1 siTR4 co-culture significantly decreased the macrophage recruitment (Figure 1C). Importantly, we found knocked-down TR4 in PCa cells also led to similar results showing decreased macrophage recruitment to the CM of PCa siTR4/THP-1 scr co-culture system (Figure 1D). In contrast, we observed a less obvious effect when we knocked-down TR4 in THP-1 cells only (Figure 1E).

Together, results from Figure 1A-E and Additional file 1: Figure S1 suggest that knocking-down TR4 in PCa cells, and not in macrophage THP-1 cells, led to suppress macrophage recruitment to the CM from PCa siTR4/THP-1 scr (or THP-1 siTR4) co-culture system.

### Reduced macrophage migration suppressed PCa invasion

**Figure 2:**
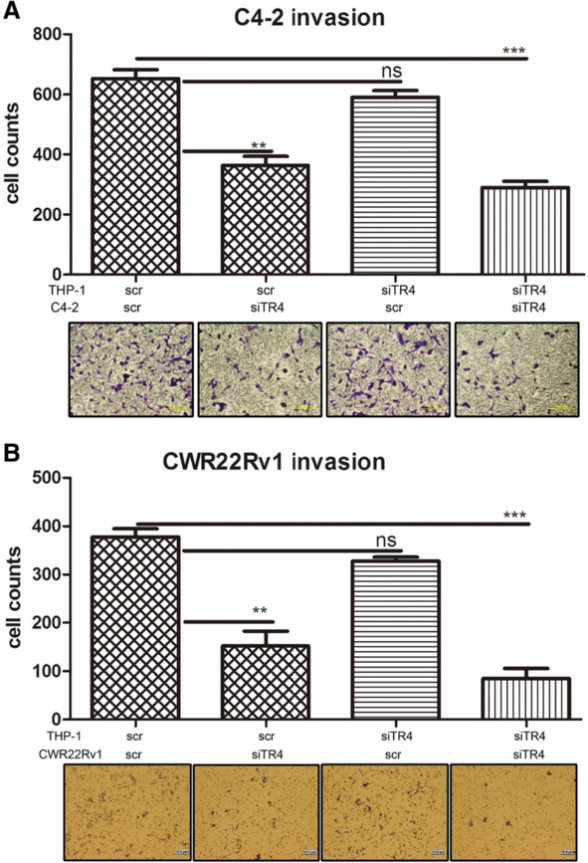
**PCa cells invasion assay under different CMs conditions.** **A**. C4-2 cells invasion assay; **B**. CWR22Rv1 cells invasion assay. Parental PCa cell invasion ability using different CMs from: PCa scr/THP-1 scr, PCa siTR4/THP-1 scr, PCa scr/THP-1 siTR4, PCa siTR4/THP-1 siTR4 (left to right). Lower pictures are images and upper columns are cell counts.

To determine the consequences after suppression the recruited macrophages, we applied co-culture system with 8 μM transwell plates to measure the invasion ability of PCa parental cells under different CMs. The CMs were prepared as in Figure 1 and put into the lower chambers of the transwell plates. The parental C4-2 or CWR22Rv1 cells were placed on the upper chambers with membranes pre-coated with matrigel for the PCa invasion assay (Figure 1A, lower and right). As shown in Figure 2A-B, we found the CM from the PCa siTR4/THP-1 siTR4 co-cultured cells significantly decreased the PCa invasion in both C4-2 (Figure 2A) and CWR22Rv1 (Figure 2B) cells tested. Similarly, CM from knocking-down TR4 in PCa cells only could also suppress PCa cells invasion. In contrast, we found no significant effect when we knocked-down TR4 in only THP-1 cells (Figure 2).

Together, results from Figures 1 and 2 demonstrated that knocked-down TR4 in PCa cells might reduce the macrophage infiltration to PCa cells to suppress PCacells invasion.

TIMP-1/MMP2/MMP9 signals was altered significantly in the parental PCa cells treated with the CM from PCa siTR4/THP-1 siTR4 co-cultured system

**Figure 3:**
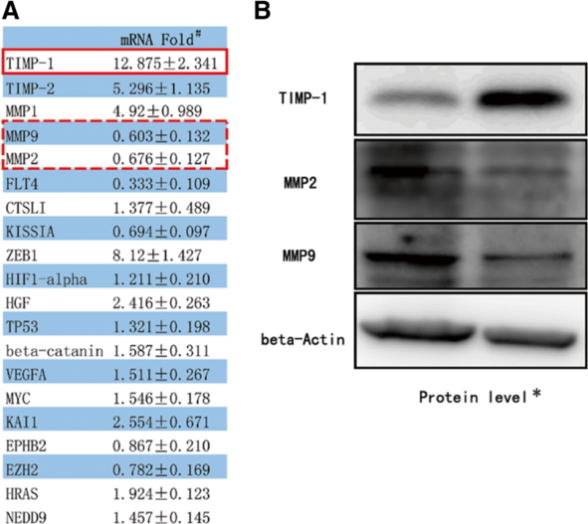
**TIMP-1/MMP2,9 singaling showed obvious change when parental C4-2 cells cultured in the CM from THP-1 siTR/C4-2 siTR4.** **A**: mRNA level of qPC Rarray; **B**: Protein level of TIMP-1/MMP2/MMP9 signaling. * Parental C4-2 cells were treated for 24 hours with the CMs from the co-culture of PCascr/THP-1 scr (control) and C4-2 siTR4/THP-1 siTR4 (study). mRNA was isolated and then metastasis-related genes qPCR array were applied to screen for the potential factors in knocked-down of TR4-reduced macrophage recruitment-PCa cells invasion. These folds represent the ratio of study/control. # Western blotting to confirm the change of TIMP-1/MMP2/MMP9 signaling. Left line is control group, right line is study group.

To dissect the potential molecular mechanism why targeting TR4 could suppress PCa invasion *via* suppression of macrophage recruitment, we screened tumor metastasis-related genes using qPCR gene microarray. We found that the TIMP-1 mRNA expression increased in the parental PCa cells treated with the CM from PCa siTR4/THP-1 siTR4 co-culture system when compared with the CM from PCa scr/THP-1 scr co-culture system. Interestingly, the expression of other two metastasis-related genes (MMP2 and MMP9) was decreased (Figure 3A). Western blotting also confirmed the altered protein expression of TIMP-1/MMP2/MMP9. 9 (Figure 3B). As TIMP-1 could function as suppressor for MMP2 and MMP9 [24,25], TIMP-1 to MMP2/MMP9 signals may be the key factor to reduce the parental PCa cells invasion treated with the CM from PCa siTR4/THP-1 siTR4 co-culture system.

### TIMP-1 antibody reversed the knocked-down TR4 effect on PCa invasion

**Figure 4:**
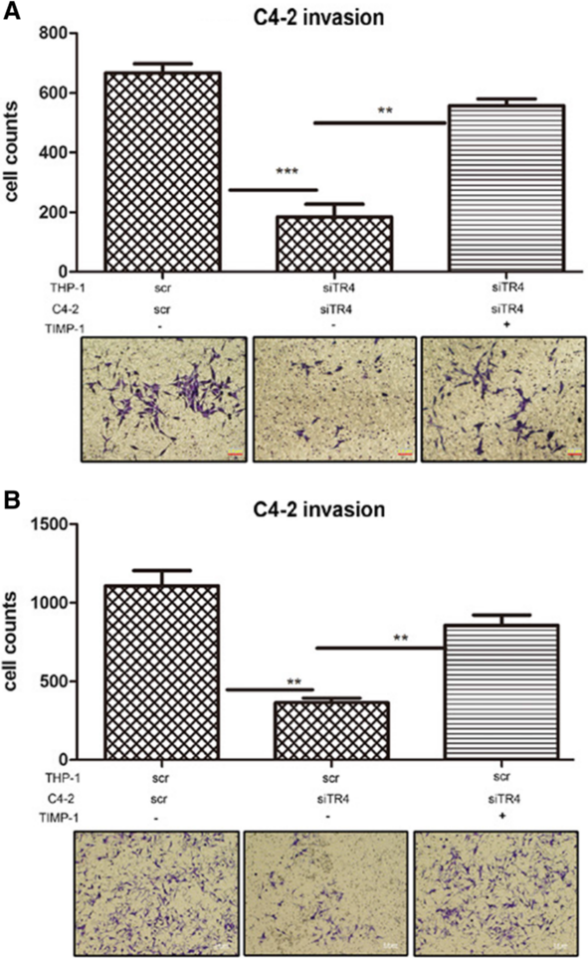
**Neutralization study with TIMP-1 antibody.** TIMP-1 neutralization antibody or IgG, as vehicle control, were added into the CMs in lower chamber to study PCa cells invasion ability. **A**. CM: PCa siTR4/THP-1 siTR4; **B**. CM: PCa siTR4/THP-1 scr.

To confirm the results above, we then applied an interruption assay to see if blocking TIMP-1 to MMP2/MMP9 signals with neutralizing anti-TIMP-1 antibody might alter the TR4 ability to influence PCa invasion. As shown in Figure 4, addition of neutralizing anti-TIMP-1 antibody reversed the TR4 ability to alter the C4-2 invasion. (Figure 4A: CM from C4-2 siTR4/THP-1 siTR4 co-culture system; Figure 4B: CM from C4-2 siTR4/THP-1 scr co-culture system). Similar results were also found when we replaced C4-2 with CWR22RV-1 (Additional file 1: Figure S2).

Together, results from Figure 3, Figure 4 and Additional file 1: Figure S2 demonstrated that TIMP-1 to MMP2/9 signals might play key roles to mediate TR4-macrophages effects on PCa cell invasion.

### Higher TR4 expression with more macrophages, lower TIMP-1, and stronger MMP2/MMP9 expression in human PCa of higher Gleason score

**Figure 5:**
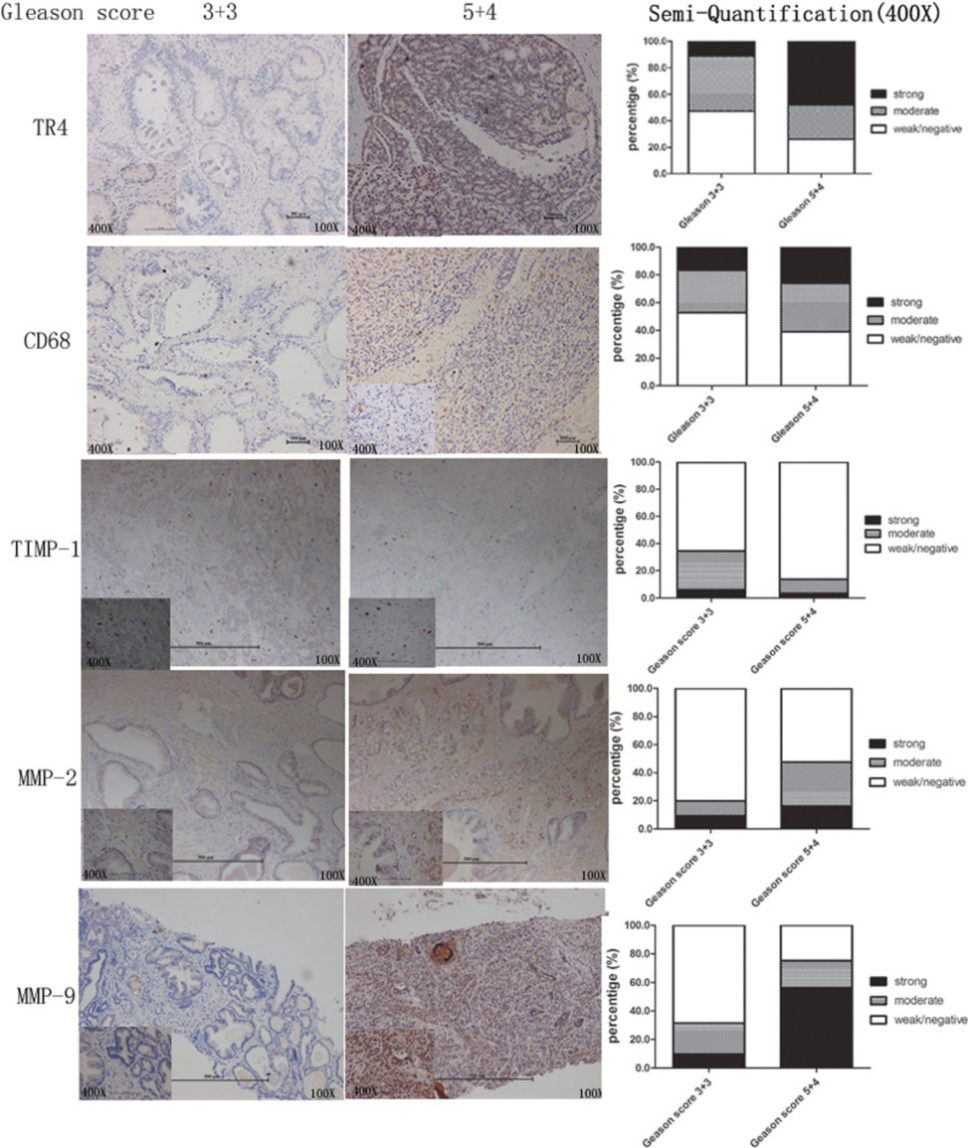
**IHC staining result investigating TR4 level and macrophage infiltration in tumor tissues of PCa patients, as well as TIMP-1/MMP2/MMP9 signaling.** IHC staining was performed using TR4 antibody (1:300) ,CD68 antibody (1:200),TIMP-1 antibody (1:200),MMP2 antibody (1:200),MMP9 antibody (1:200).

To further confirm our *in vitro* results above, we then examined the expression of TR4 and macrophage infiltration (*marker*: *CD68)* in clinical tissue samples obtained from PCa patients with different Gleason scores. We found higher TR4 expression in PCa with the Gleason score 5 + 4 patients compared with those with Gleason score 3 + 3 patients (Figure 5, first line), and more infiltrated macrophages in PCa with the Gleason score 5 + 4 patients (Figure 5, second line). Importantly, we also found lower TIMP-1 expression with higher MMP2/MMP9 expression in PCa of Gleason score 5 + 4, compared with Gleason score 3 + 3 (Figure 5, third-fifth line).

Together, results from Figures 1, 2, 3, 4 and 5 demonstrate that TR4 expression is correlated with macrophage infiltration and PCa invasion that may involve the modulation the TIMP-1-MMP2/MMP9 signaling.

## Discussion

Inflammation is a protective reaction of living tissue to internal or external environmental stimuli that aims to remove the invasive agents and restore the tissue physiology. Approximately, a quarter of tumors may attribute to chronic infections and other types of unresolved inflammation [5]. The inflammation present in the tumor microenvironment is characterized by leukocyte infiltration, ranging in size, distribution and composition, and may include the tumor-associated macrophages mast cells, dendritic cells, natural killer cells, neutrophils and lymphocytes [5-8]. Macrophages are among the first immune cells to infiltrate the tumor lesions and then influence the tumor progression/invasion via secretion of several growth factors, cytokines or chemokines, including colony stimulating factor 1 (CSF-1), vascular epithelial growth factor (VEGF) and CCL2 [3,5-8]. Recent studies also demonstrated that targeting the androgen receptor with siRNA might lead to enhance PCa invasion *via* increasing the recruitment of infiltrating macrophages that involved the modulation of CCL2-STAT3 signaling [3].

The imbalance between MMPs and their tissue inhibitors TIMPs have been implicated significantly in PCa progression, and usually the observed imbalance of MMPs-TIMPs could be due to a loss of TIMP-1 [26]. TIMP-1 may function as suppressor to inhibit several metastasis genes, such as MMP2 and MMP9. Many studies also documented well that TIMP-1 and MMP2/MMP9 could be related to the invasive/metastatic ability of PCa [26-28].

Xie *et al*. [22] reported that foam cell formation was reduced in TR4 knockout (TR4^−/−^) mice *via* manipulation of CD36 expression in cardiovascular diseases. Mahajan *et al*. [23] also found M. tuberculosis macrophage lipids could interact with TR4 to ensure survival of the pathogen by modulating macrophage function. Our current study also found an important role of TR4 in tumor associated macrophages: TR4 in PCa cells may play a positive role to attract macrophage infiltration, the consequent crosstalk between PCa cells and macrophage may produce some changes in the tumor microenvironment around PCa cells, and these changes may alter the invading ability of PCa cells via different mechanisms. We were more interested with the molecular mechanism of decreased PCa cell invasion under the CM from the co-culture system of PCa siTR4/THP-1 siTR4. The further dissection found TIMP-1 level was increased when parental PCa cells were cultured in the CM from C4-2 siTR4/THP-1 siTR4, and addition of neutralizing antibody of TIMP-1 reversed this effect. Together, these results suggest that TIMP-1-MMP2/9 signals may play key roles in the TR4-mediated macrophages infiltration-PCa cell invasion.

Other key genes related to the PCa invasion including TIMP-2, MMP1, and ZEB1 may be also involved in this macrophages-PCa co-culture system, and more detailed studies may be needed to further dissect their roles in mediating this infiltrated macrophages-PCa cell invasion.

In summary, these results suggest that targeting TR4 might suppress macrophage infiltration and consequently reduce the PCa cell invasion. Further studies *via* development of small molecules to target TR4 may help us to develop a potential new therapeutic approach to suppress this newly identified signal to better suppress the PCa at later metastatic stages.

## Methods

### Cell culture

Two human PCa cell lines C4-2, CWR22RV-1, and the human THP-1 cell line derived from leukemia cells were purchased from the American Type Culture Collection. PCa cells were maintained in RPMI 1640 (CWR22Rv-1) or DMEM (C4-2) containing 10% FBS, THP-1 cells were maintained in RPMI1640 medium containing 10% heat-inactivated FBS, and 5 mM 2-mercaptoethanol. All media contained 2 mM L-glutamine, 100 IU/ml penicillin and 100 μg/ml streptomycin and all cells were incubated at 37°C in a humidified incubator at 5% CO2.

### Antibodies

Anti-TR4 (PP-0107B-00) and anti-TIMP-1 (AF970) antibodies were purchased from R&D systems (Minneapolis, MN). Anti-GAPDH (6c5) antibody was purchased from Santa Cruz Biotechnology (Santa Cruz, CA). CD68 antibody (ab125212) was purchased from Abcam (Cambridge, MA).

### Reverse transcription (RT) and qPCR analysis

Total RNAs were isolated from C4-2, and CWR22Rv-1 cells using TRIzol® Reagent (Invitrogen). RNA (2 μg) was used as a template for the RT reaction (20 μl system). The RT reaction mixture (Invitrogen) contained 1 μl (10 pM) of primers. The resultant cDNA was then used to carry out real time quantitative RT-PCR, using SYBR Green PCR MasterMix, on the iCycleriQ™ PCR cycler and detection system (Bio-Rad Laboratories, Hercules, CA). PCR conditions were as follows: 95°C for 5 min, followed by 40 cycles at 95°C for 1 min, 55°C for 1 min and 72°C for 1 min. The final extension was at 72°C for 5 min. Calculation of relative gene expression was performed using the 2^-ΔΔ^CT method.

### THP-1 recruitment assay

The PCa/macrophage cells were co-cultured in 0.4 μM pore size transwell plates for 24 hours. Then the conditioned media (CM) were collected, diluted with normal culture media, plated into the lower chamber of new 24-well transwells with 5 μM pore polycarbonate membrane insert (Corning, #3422, BD Biosciences, San Jose, CA). 1×10^5^ of parental THP-1 cells were plated onto the upper chamber for macrophage migration assay. The cells migrated into the lower chamber were measured using 3-(4,5-Dimethylthiazol-2-yl)-2,5-diphenyltetrazolium bromide (MTT)method. MTT solution (100 mL, 5 mg/mL in PBS) was added to each well of the transwell plates and incubated for 2 hours at 37°C. Centrifuged at 3000 rmp for 20 minutes, discarded the supernatant discarded and 300 μl/well dimethylsulfoxide (DMSO) was added to dissolve the formazan. The absorbance was immediately measured at 570 nm using a Bio-Rad Absorbance Reader. Each sample was assayed in triplicate and the experiments were repeated at least three times.

### Cell invasion assay

CM was collected and diluted as above and added into the lower chamber of the 24-well transwell with 8 μM pore polycarbonate membrane insert (Corning, #3422) pre-coated with matrigel for cell invasion assay. The parental PCa cells were plated onto upper chamber at 1×10^5^. Each sample was assayed in triplicate. The cells invaded to the lower chamber were fixed, stained using 1% toluidine blue, and the numbers averaged after counting 6 randomly selected fields. Each experiment was repeated at least in triplicate.

### Western blotting

The cells were washed with 1xPBS and scraped into a lysis buffer containing the proteinase inhibitor cocktail (Roche). Protein concentrations were measured with the BCA protein reagent (Pierce Chemical, Rockford, IL). Approximately 50 μg of protein/lane were loaded and run on the polyacrylamide gel with a Tris/glycine running buffer system and then transferred onto a polyvinylidene difluoride membrane. The primary antibodies with dilutions of 1:500 to 1:1,000 were added and then incubated overnight in the cold room with vigorous shaking. The horseradish peroxidase-conjugated secondary antibody (Pierce Chemical, Rockford, IL) was added and the signals were detected by adding the enhanced chemiluminescence Western blotting detection reagents (Amersham Biosciences, Piscataway, NJ).

### Clinical samples and immunohistochemical (IHC) staining

Clinical tissues of twenty-three PCa patients of Gleason score 5 + 4 and thirty-six Gleason score 3 + 3 PCa patients were collected from Sir Run Run Shaw Hospital. Tissues were fixed in 10% (v/v) formaldehyde in PBS, embedded in paraffin, and cut into 4 μm sections and used for IHC staining with human TR4/CD68 antibodies. To enhance antigen exposure, the slides were treated with 1 × EDTA at 98°C for 10 min for antigen retrieval. The slides were incubated with endogenous peroxidase blocking solution to inhibit endogenous peroxidase, and then were incubated with the primary antibody at room temperature for 60 min. After rinsing with Tris-buffered saline, the slides were incubated for 45 min with biotin-conjugated secondary antibody, washed, and then incubated with enzyme conjugate horseradish peroxidase (HRP)-streptavidin. Freshly prepared DAB (Zymed, South San Francisco, CA) was used as substrate to detect HRP. Finally, slides were counterstained with hematoxylin and mounted with aqueous mounting media. All the tissues were stained and examined by 2 independent pathologists in the same hospital in a blinded manner without prior knowledge of the clinical information. German Immunoreactive Score (IRS)was calculated to measure the protein levels. Briefly, the IRS assigns sub-scores for the percentage of immunoreactive cells (0–4) and immunoreactive intensity (0–3), then multiplies them to yield the IRS score, which ranged from 0 to 12. The percent positivity was scored as “0” (<1%), “1” (1-10%), “2” (11-50%), “3” (51-80%), “4” (>80%). The staining intensity was scored as “0” (negative), “1” (weak), “2” (moderate), and “3” (strong). Scores were considered negative (0–1), weakly positive (2–4), moderately positive (6–8), and strongly positive (9–12).

### Statistics

Data are presented as the means ± SD for the indicated number of separate experiments. The statistical significance of differences between two groups of data was analyzed by paired t-test and P-values < 0.05 were considered significant.

## Additional file

Additional file 1: Figure S1. The effect of CMs on macrophage recruitment. The C4-2 (5 × 10^5^)/THP-1 (5 × 10^5^) cells were co-cultured in 0.4 μM pore size transwell plates. 1 × 10^6^ C4-2 only or THP-1 only were inoculated into a 6-well plate. CMs of these three groups were collected after 24 hours culture. The CMs were diluted with freshmedia at 1:1 ratio then plated into the lower chamber of 24-well transwells with 5 μM pore size. 1x10^5^parental THP-1 cells were plated onto the upper chamber for macrophage migration assay. **Figure S2.** Neutralization study with CWR22Rv-1. TIMP-1 neutralization antibody or IgG, as vehicle control, were added into the CMs in lower chamber to observe CW22Rv-1 cells invasion ability.
