# Correction to: Targeting TR4 nuclear receptor suppresses prostate cancer invasion via reduction of infiltrating acrophages with alteration of the TIMP-1/MMP2/MMP9 signals

**DOI:** 10.1186/s12943-020-01212-7

**Published:** 2020-05-30

**Authors:** Xianfan Ding, Dong-Rong Yang, Liqun Xia, Bide Chen, Shicheng Yu, Yuanjie Niu, Mingchao Wang, Gonghui Li, Chawnshang Chang

**Affiliations:** 1 Department of Urology and Chawnshang Chang Liver Cancer Center, Sir Run Run Shaw Hospital, School of Medicine, Zhejiang University, Hangzhou, 310016 China; 2 George Whipple Lab for Cancer Research, Departments of Pathology, Urology and Radiation Oncology, and The Wilmot Cancer Center, University of Rochester Medical Center, Rochester, NY 14646 USA; 3 Department of Urology, the 2nd Affiliated Hospital of Soochow University, Suzhou, 215004 China; 4 Chawnshang Chang Sex Hormone Research Center, Tianjin Institute of Urology, Tianjin Medical University, Tianjin, 300211 China; 5 Sex Hormone Research Center, China Medical University/Hospital, Taichung, 404 Taiwan

**Correction to: Mol Cancer (2015) 14:16**

**https://doi.org/10.1186/s12943-014-0281-1**

Following the publication of the original article [1], the corresponding author recently found error in the Fig. 2A. Below is the corrected image. This error could be due to mislabel and/or incorrect insertion during transfer of numerous images between computers in two countries after he returned from USA and became occupied with his university work to establish a laboratory. They assure that the correction of this image will not alter the conclusion of the results. The authors are appreciative for the publication of this correction paper.


