# Supplementary figures and images for: Targeting TR4 nuclear receptor suppresses prostate cancer invasion via reduction of infiltrating macrophages with alteration of the TIMP-1/MMP2/MMP9 signals
Source: Mol Cancer. 2015 Jan 27;14(1):16. doi: 10.1186/s12943-014-0281-1 (PMC4316804; doi:10.1186/s12943-014-0281-1)

Supplemental Figure 1

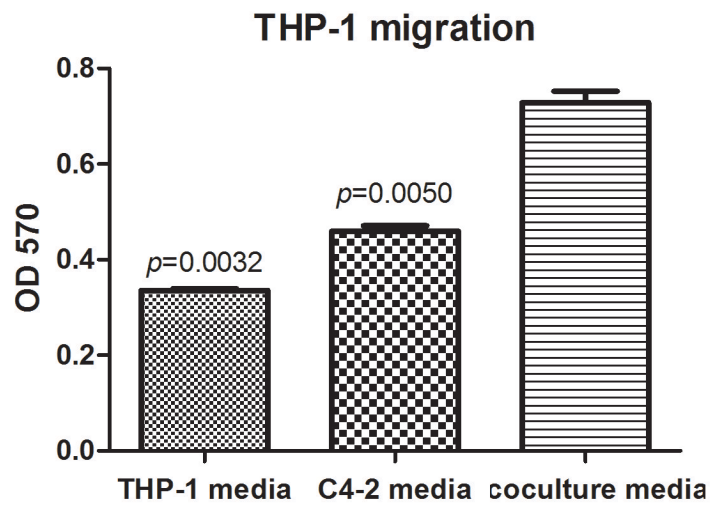

Supplemental Figure 2

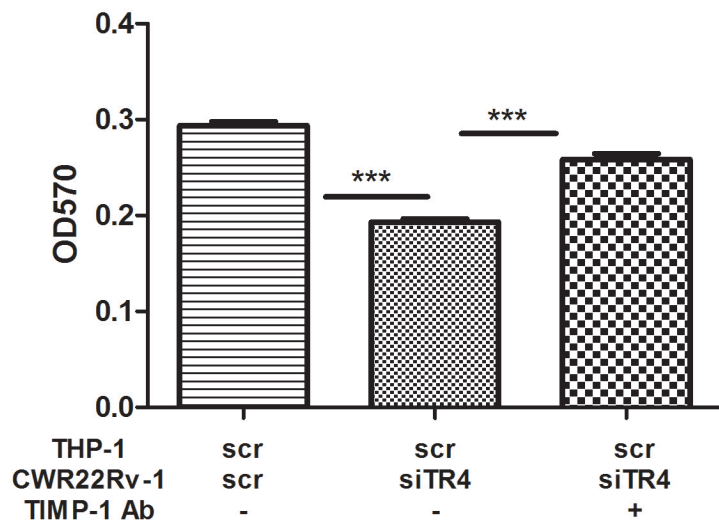

Supplement: Additional file 1: Figure S1. — The effect of CMs on macrophage recruitment. The C4-2 (5 × 10^5^)/THP-1 (5 × 10^5^) cells were co-cultured in 0.4 μM pore size transwell plates. 1 × 10^6^ C4-2 only or THP-1 only were inoculated into a 6-well plate. CMs of these three groups were collected after 24 hours culture. The CMs were diluted with freshmedia at 1:1 ratio then plated into the lower chamber of 24-well transwells with 5 μM pore size. 1x10^5^parental THP-1 cells were plated onto the upper chamber for macrophage migration assay. Figure S2. Neutralization study with CWR22Rv-1. TIMP-1 neutralization antibody or IgG, as vehicle control, were added into the CMs in lower chamber to observe CW22Rv-1 cells invasion ability. [file 12943_2014_281_MOESM1_ESM.pdf]
